# Bubble-test for detection of a patent foramen ovale in young to middle-aged ischemic stroke patients

**DOI:** 10.3389/fneur.2026.1743561

**Published:** 2026-05-11

**Authors:** Anna Bogdanova, Lena Mers, Alexander Sekita, Laura Besendorf, Ludwig Singer, Tobias Engelhorn, Caroline Merkel, Kosmas Macha, Arnd Doerfler, Stefan Schwab, Stefan T. Gerner

**Affiliations:** 1 Department of Neurology, University Hospital Erlangen, Erlangen, Germany; 2 Department of Neuroradiology, University Hospital Erlangen, Erlangen, Germany; 3 Department of Cardiology, University Hospital Erlangen, Erlangen, Germany

**Keywords:** bubble test, cardiac, cryptogenic stroke, ischemic stroke, patent foramen ovale, sonography, young stroke patients

## Abstract

**Background:**

Current stroke guidelines do not recommend routine screening for a patent foramen ovale (PFO) in patients with acute ischemic stroke (AIS) of undetermined cause. This study evaluated the diagnostic accuracy of the transcranial Doppler bubble test (TCD-bubble) for PFO detection in younger AIS patients.

**Patients and methods:**

Data from a prospective institutional stroke registry were retrospectively analyzed, including AIS patients aged ≤65 years treated with intravenous thrombolysis (IVT) and/or endovascular therapy (EVT) over 5 years. Right-to-left shunt (RLS) was assessed using the TCD-bubble test. Sensitivity, specificity, positive predictive value (PPV), and negative predictive value (NPV) for PFO detection were calculated using transesophageal echocardiography (TEE) as the reference. Furthermore, we included a ROC-analysis to provide a bubble-threshold and calculated a new sensitivity, specificity, PPV and NPV.

**Results:**

Of 383 patients, 120 (31%) underwent TCD-bubble testing: 61 (50.8%) had no RLS, 20 (16.7%) had RLS only during the Valsalva maneuver, and 39 (32.5%) had RLS at rest. Cardiovascular risk profiles and functional outcomes did not differ between RLS-positive and -negative patients. Among 87 patients with TEE, PFO was confirmed in 69.4% of RLS-positive cases (PPV), while a negative TCD-bubble test excluded PFO in 97.4% (NPV). Sensitivity and specificity were 97.1 and 71.2%, respectively. A cut-off of ≥3 bubbles in TCD-bubble testing resulted in a sensitivity of 91.4% and specificity of 88.5% for PFO detection.

**Conclusion:**

The TCD-bubble test is a reliable, non-invasive screening tool for PFO in younger AIS patients with high sensitivity and NPV for PFO detection. A positive result warrants confirmatory TEE, while a negative test may preclude further invasive evaluation.

## Introduction

Ischemic stroke remains the second leading cause of death worldwide and a major contributor to long-term disability (1). Among patients aged ≤65 years, 20–30% of ischemic strokes remain cryptogenic despite comprehensive diagnostic evaluation, highlighting the need for refined diagnostic strategies (2, 3).

A PFO is implicated in up to one-half of cryptogenic strokes in this age group (4). Randomized controlled trials have demonstrated that PFO closure can reduce the risk of recurrent stroke in selected patients, underscoring the importance of reliable detection (5, 6). However, identifying suitable candidates remains challenging, as the current reference standard—a TEE—is semi-invasive, requires sedation, and carries procedural risks (7).

The TCD-bubble test is a non-invasive, ultrasound-based technique for detection of right-to-left shunts by visualizing microbubbles in the cerebral circulation after intravenous injection of agitated saline. Its bedside feasibility and high sensitivity make it a promising first-line screening tool in suspected PFO. Nonetheless, reported diagnostic accuracy varies across studies, and the clinical significance of shunt detection at rest versus only during Valsalva maneuver remains uncertain (8).

This retrospective study analyzes the diagnostic value of the TCD-bubble test in patients ≤65 years with AIS who underwent reperfusion therapy. We evaluated the diagnostic performance against TEE and examined clinical characteristics associated with RLS and test utilization. The aim was to clarify the role of the TCD-bubble test in the diagnostic algorithm for suspected PFO in young to middle aged AIS-patients.

## Methods

### Study design and participants

This study is representing a retrospective subanalysis of our prospective local stroke registry STAMINA over a 5-year period. In summary, the STAMINA registry (Stroke Research Consortium in Northern Bavaria, www.clinicaltrials.gov, NCT04357899), included AIS patients treated with either IVT, EVT or both at the Department of Neurology, University Hospital Erlangen, Germany. The original prospective local stroke registry was designed to collect epidemiological diagnostic and therapeutic data of patients receiving IVT, EVT or both. For our subanalysis we excluded patients aged 65 years or older. The study received approval from the local ethics committee of Friedrich-Alexander University Erlangen-Nuremberg, Germany (Registration No. 62_21B).

### Parameter selection

Baseline characteristics included demographic data (i.e., age, sex), functional status upon admission based on the modified Rankin Scale (mRS), cardiovascular risk factors, and treatment characteristics (IVT, EVT, time metrics). Stroke characteristics comprised neurological status at admission using the National Institutes of Health Stroke Scale (NIHSS) and the Glasgow Coma Scale (GCS), as well as stroke etiology according to the TOAST-classification. Functional outcome was assessed at discharge and after 90d via semiquantitative telephone-interview using the mRS.

### TCD-bubble test and TEE protocol

At our stroke unit, TCD-bubble testing was routinely considered in patients aged ≤65 years with AIS treated with IVT and/or EVT within 3 days of admission. Specifically, TCD-bubble testing was performed in the absence of known atrial fibrillation, symptomatic intra- or extracranial large-artery stenosis, or other clearly established stroke mechanisms (i.e., vasculitis, dissection, coagulopathy, air embolism). In addition, patients had to be non-intubated and clinically stable to allow standardized examination. Patients were excluded from TCD-bubble testing in cases of persistent intubation, reduced consciousness, severe clinical instability, early discharge or transfer, or when a competing stroke etiology had already been established.

The TCD-bubble examination was performed by certified neurologists before transesophageal echocardiography was scheduled and therefore without knowledge of the subsequent TEE findings.

The middle cerebral artery was insonated using an ACUSON Sequoia Select (Siemens Healthineers) (for detailed procedure see Supplementary methods). A contrast suspension was injected via an antecubital vein. Injections were performed (1) at rest and (2) 4–8 s before a standardized Valsalva maneuver, with ≥1 min between injections. Microembolic signals (“bubble hits”) were recorded continuously, and the number and temporal distribution were documented. RLS was scored according to the following criteria:

- “Clear evidence of RLS” was defined as detection of bubbles at rest or >20 bubbles during the Valsalva maneuver.
- “Minor evidence of RLS” was defined as detection of ≤20 bubbles during the Valsalva maneuver only.

All patients with positive TCD-bubble test were planned for a standardized TEE within 24 h, performed by board-certified cardiologists who were routinely informed about the clinical indication for the examination, including prior non-invasive test results, and were therefore not systematically blinded to the TCD-bubble findings. TEE confirmed PFO presence and assessed shunt size, atrial septal aneurysm, and shunt flow parameters (for detailed procedure and exclusion criteria see Supplementary methods) (5, 6).

The final etiological classification was performed independently by a stroke unit physician based on all available clinical, imaging, and diagnostic information, including both TCD and TEE results, in accordance with routine clinical workflows.

### Statistical analyses

All statistical analyses were performed using IBM SPSS Statistics v28.0.^1^ Categorical variables were compared with Pearson’s *χ*^2^ or Fisher’s exact test. Non-normally distributed continuous variables were analyzed with the Mann–Whitney U test. A two-sided *p*-value <0.05 was considered statistically significant. Results are presented as absolute and relative frequencies or as medians with interquartile ranges (IQR). Diagnostic accuracy of the TCD-bubble test was assessed by calculating sensitivity, specificity, positive (PPV) and negative predictive value (NPV) using TEE as the reference standard. Receiver operating characteristic (ROC) analysis was performed independently using the original, uncategorized bubble counts recorded during the TCD-bubble examination as an ordinal/continuous variable to identify the optimal bubble count cut-off for PFO prediction. No prior dichotomization or categorization was applied before the ROC analysis.

## Results

**Figure 1:**
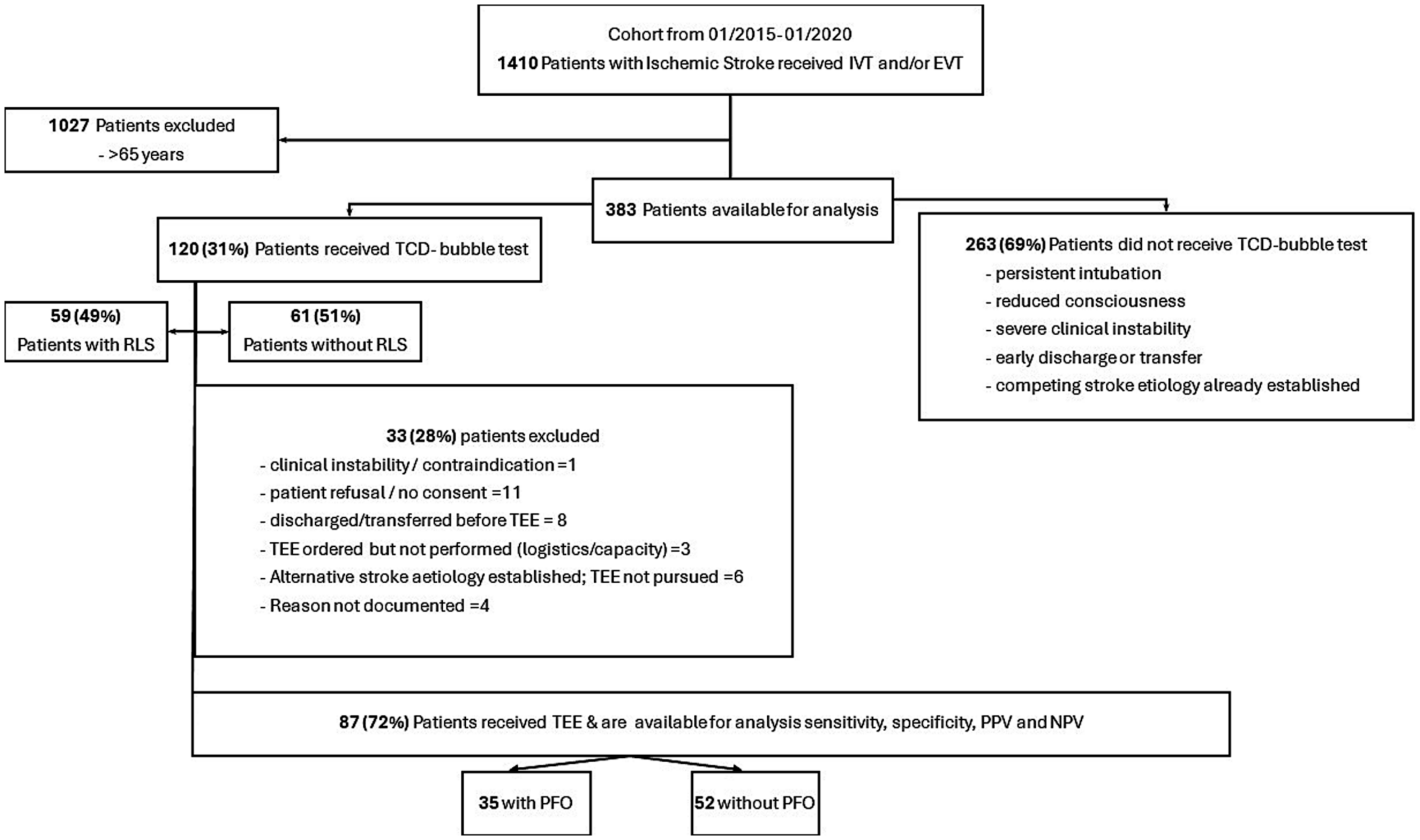
Flow chart of study participants. Overall, data of 1,410 patients with AIS receiving either IVT, EVT, or both were enrolled between 01/2015 and 01/2020. AIS, acute ischemic stroke; IVT, intravenous thrombolysis; EVT, endovascular treatment; TCD, transcranial Doppler; RLS, right–left shunt.

Over a 5-year period, 1,410 AIS-patients underwent reperfusion therapy at our center (see Figure 1). After excluding 1,027 patients aged > 65 years, 383 patients remained for analyses. Of those, 120 (31%) underwent TCD-bubble testing and 263 (69%) did not. Patients tested with TCD-bubble were younger (52 vs. 57 years) and had less premorbid disability compared to those without testing (mRS > 1: 14% vs. 27%). Admission NIHSS was lower in the TCD group (5 vs. 11) and EVT less frequently performed (39% vs. 62%), whereas IVT rates were comparable. Stroke etiology differed significantly, with a higher proportion of undetermined or competing TOAST etiologies in the TCD-bubble group (50% vs. 31%). Functional outcomes were better in the TCD-bubble group, with lower median NIHSS scores at discharge (1 vs. 4) (Table 1). Of 120 patients undergoing TCD bubble testing, 83 (69%) received TEE as the reference standard and 4 (3%) had an already diagnosed PFO with prior TEE. To address potential selection bias, we compared baseline characteristics between patients who did and did not undergo TEE. Apart from higher age and a higher prevalence of arterial hypertension in the non-TEE group, no clinically meaningful differences were observed in demographic characteristics, stroke severity, stroke etiology distribution, acute treatment, or functional outcomes (Supplementary Table 2).

**Table 1:** Comparison of AIS patients who received or did not receive TCD-Bubble Test.

AIS-patients ≤65a treated with IVT/EVT (*n* = 383)	Received TCD-bubble test (*n* = 120)	Did not receive TCD-bubble test (*n* = 263)	*p*-value
Age, y; median (IQR)	52 (48–58)	57 (52–61)	0.001
Female sex; *n* (%)	37 (31%)	101 (38%)	0.19
Prior medical history; *n* (%)			
Premorbid mRS; median (IQR)	0 (0–0)	0 (0–1)	0.008
Premorbid disability, i.e., mRS ≥ 1, no (%)	17 (14%)	70 (27%)	0.006
Alcohol abuse	14 (12%)	51 (19%)	0.071
Nicotine abuse	58 (49%)	114 (43%)	0.39
Hypertension	53 (44%)	165 (63%)	0.13
Diabetes mellitus Type II	17 (14%)	54 (21%)	0.16
Hypercholesterolemia	69 (58%)	135 (51%)	0.36
Coronary artery disease	12 (10%)	39 (15%)	0.24
Atrial fibrillation	6 (5%)	41 (16%)	0.006
Renal failure	10 (8%)	35 (13%)	0.20
Stroke characteristics			
NIHSS on admission; median (IQR)	5 (3–5)	11 (6–17)	0.001
GCS on admission: median (IQR)	15 (13–15)	14 (10–15)	0.001
TOAST classification; *n* (%)			0.001
Microangiopathy	12 (10%)	32 (13%)	
Macroangiopathy	11 (9%)	59 (23%)	
Cardioembolic	11 (9%)	57 (22%)	
Unknown	60 (50%)	82 (31%)	
Other	25 (21%)	32 (31%)	
Endovasular thrombectomy; *n* (%)	47 (39%)	162 (62%)	0.001
Intravenous thrombolysis; *n* (%)	105 (88%)	217 (83%)	0.22
Outcome scores			
NIHSS at discharge; median (IQR)	1 (0–4)	4 (1–10)	0.001
mRS 90 days; median (IQR)	2 (1–2)	2 (2–4)	0.001

AIS, acute ischemic stroke; IVT, intravenous thrombolysis; IQR, interquartile range; mRS, modified Rankin Scale; NIHSS, National Institutes of Health Stroke Scale; GCS, Glascow Coma Scale.

### Diagnostic performance of TCD-bubble

**Figure 2:**
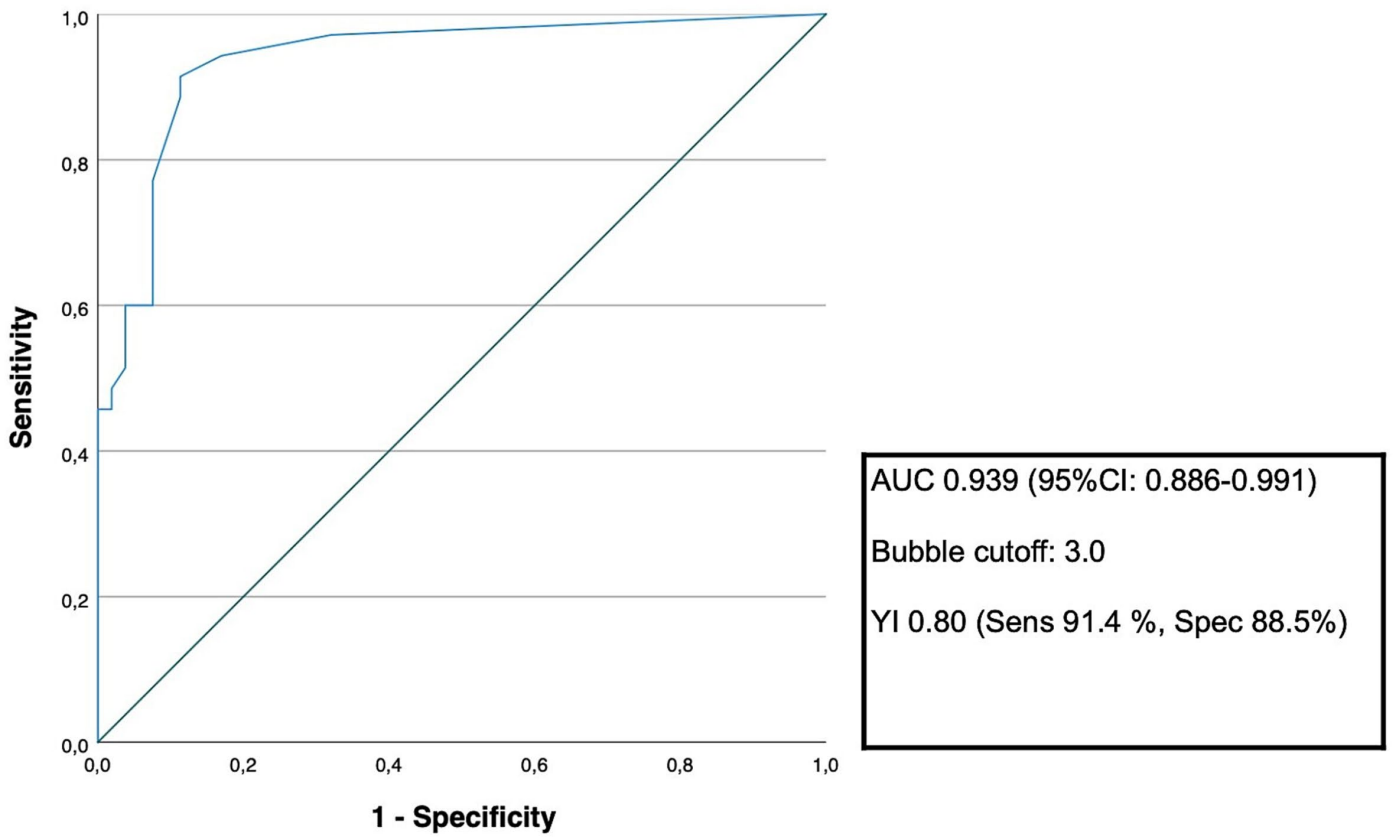
ROC-analysis for optimal cut-off number of bubbles in the TCD-bubble test. The receiver operating characteristic (ROC) analysis was conducted to assess the best cut-off number of bubbles in the TCD-bubble test to maximize the sensitivity and specificity for the prediction of a PFO. The area under the curve (AUC) with the corresponding 95% confidence interval as well as the Youden-index with sensitivity and specificity is presented.

Of 87 patients who underwent both TCD-bubble and TEE, PFO was confirmed in 34/49 RLS-positive cases (sensitivity = 97.1%; 95% CI 85.1–99.9%) and excluded in 37/38 RLS-negative cases (NPV = 97.4%; 95% CI 84.2–99.6%). Specificity was 71.2% (95% CI 56.9–82.9%) and PPV 69.4% (95% CI 59.6–77.7%) (Table 2). Additionally, we performed a ROC-analysis to identify the best cut-off value of detected bubbles in the TCD-bubble test for detection of probable PFO (Figure 2). A cut-off of ≥3 bubbles yielded sensitivity of 91.4% and specificity of 88.5% for PFO detection (AUC = 0.94; 95% CI 0.89–0.99; Table 2; Figure 2).

**Table 2:** Performance of TCD-Bubble Test for the detection of underlying PFO (A-1) & (A-2) vs. with a cut-off of 3 bubbles (B-1) & (B-2).

(A-1) Diagnostic performance without application of a bubble-count threshold: contingency table			
	RLS	No RLS	Total
PFO, No. (%)	34 (40%)	1 (1%)	35 (40%)
No PFO, No. (%)	15 (17%)	37 (43%)	52 (60%)
Total, No. (%)	49 (56%)	38 (44%)	87 (100%)

(A-2) Diagnostic performance without application of a bubble-count threshold: accuracy measures	
	Value (95% CI)
Sensitivity	97.14% (85.08–99.93%)
Specificity	71.15% (56.92–82.87%)
Positive predictive value	69.39% (59.57–77.71%)
Negative predictive value	97.37% (84.20–99.61%)

(B-1) Diagnostic performance using a cut-off of ≥3 bubbles: contingency table			
	Number bubbles <3	Number bubbles ≥ 3	Total
PFO, No. (%)	3 (3%)	32 (36%)	35 (40%)
No PFO, No. (%)	46 (53%)	6 (7%)	52 (60%)
Total, No. (%)	49 (56%)	38 (44%)	87 (100%)

(B-2) Diagnostic performance using a cut-off of ≥3 bubbles: accuracy measures	
	Value (95% CI)
Sensitivity	91.43% (76.94–98.20%)
Specificity	88.46% (76.56–95.65%)
Positive predictive value	84.21% (71.39–91.93%)
Negative predictive value	93.88% (83.80–97.85%)

Table A: Performance without bubble-count threshold (any RLS considered positive).

1: 2*2 Table showing the contingency (cross-tabulation of TCD results vs. confirmed PFO status).

2: Table with calculated sensitivity, specificity, PPV & NPV with 95% CI.

Table B: Performance using a cut-off of ≥3 bubbles to define a positive test.

1: 2*2 Table for the contingency (cross-tabulation of TCD results vs. confirmed PFO status).

2: Table with calculated sensitivity, specificity, PPV & NPV with 95% CI.

RLS, right–left-shunt; PFO, patent foramen ovale; CI, confidence interval; PPV, positive predictive value; NPV, negative predictive value.

### Prevalence and characteristics of RLS

In the TCD-bubble group, 59/120 patients (49.2%) had a RLS. Of these, 20 (34%) were positive only during the Valsalva maneuver and 39 (66%) at rest.

Neither baseline characteristics, the premorbid mRS nor neurological scores on admission and follow-up varied significantly between the groups with RLS under Valsalva and RLS in rest. Median bubble count was similar between subgroups (rest: 15, Valsalva only: 17). Approximately half of the patients had 1–20 bubbles, and up to 20% reached the maximum count of 99. 12 patients had a bubble count of 1–2 (5 during Valsalva, 7 in rest) (Table 3). RLS prevalence was highest in patients aged 40–55 years (47%), with detection at rest (31%) more frequent than during the Valsalva maneuver (16%) (Supplementary Figure 1; Table 3).

**Table 3:** Prevalence and characteristics of RLS detection during the bubble-test.

AIS-patients ≤65a with bubble test (*n* = 120)	RLS under Valsalva (*n* = 20)	RLS in rest (*n* = 39)	*p*-value
Age, y; median (IQR)	53 (38–57)	54 (45–58)	0.90
Female sex; *n* (%)	6 (30%)	11 (28%)	0.89
Body weight; median (IQR)	90 (70–96)	80 (75–91)	0.40
Premorbid mRS; median (IQR)	0 (0–0)	0 (0–0)	0.42
Stroke characteristics			
NIHSS on admission; median (IQR)	4 (2–9)	5 (4–12)	0.19
GCS on admission: median (IQR)	15 (15–15)	15 (13–15)	0.18
Arteriosclerosis in Duplex Sonography; *n* (%)	14 (70%)	19 (49%)	0.35
Number of Bubbles: median (IQR)	17 (3–30)	15 (5–59)	0.76
Outcome Scores			
NIHSS at discharge; median (IQR)	1 (0–4)	1 (0–3)	0.80
mRS 90 days; median (IQR)	2 (1–2)	1 (1–1)	0.51
Number of bubbles detected; *n* (%)			
Median (IQR)	17 (3–30)	15 (5–59)	0.76
1–20	11 (55%)	23 (59%)	
1–2	5 (25%)	7 (18%)	
3–10	2 (10%)	10 (26%)	
11–20	4 (20%)	6 (15%)	
>20	9 (45%)	16 (41%)	
Maximum: 99	3 (15%)	8 (21%)	

AIS, acute ischemic stroke; IQR, interquartile range; mRS, modified Rankin Scale; NIHSS, National Institutes of Health Stroke Scale; GCS, Glascow Coma Scale.

### Comparison among patients according to RLS-status (RLS vs. no RLS)

Baseline demographics and cardiovascular risk factors did not differ between RLS-positive and RLS-negative patients. Stroke etiology distribution was significantly different with cardioembolic and large-artery strokes more frequent in the no-RLS group. Outcomes were more favorable in RLS-positive patients, with lower NIHSS at discharge and better 90-day mRS (Supplementary Table 1). Furthermore, the distribution of mRS scores differed markedly between patients with and without RLS.

In the no-RLS group (*n* = 61), 46 patients (75%) achieved a favorable functional outcome (mRS 0–2), whereas 15 patients (25%) had an unfavorable outcome (mRS 3–6). In contrast, in the RLS group (*n* = 59), 54 patients (92%) achieved a favorable outcome (mRS 0–2), and only 5 patients (8%) had an unfavorable outcome (mRS 3–4) (Supplementary Figure 2).

## Discussion

In this retrospective study of AIS patients aged ≤65 treated with reperfusion therapy, RLS were detected in approximately one of two cases using TCD-bubble testing, consistent with reported prevalence rates of 40–50% in younger cryptogenic stroke populations (9). Patients with RLS showed lower atherosclerotic burden and more favorable functional outcomes. Consistently, patients receiving TCD-bubble testing showed a predominance of unknown or competing TOAST etiologies and fewer established cardioembolic or macroangiopathic causes, supporting targeted PFO evaluation despite high rates of IVT and EVT (10, 11). Several aspects deserve further attention.

Our results are broadly consistent with findings from similar studies, such as those by Mayerhofer et al. (12). Apart from methodological differences—most notably our additional focus on distinguishing between Valsalva-induced shunting and bubble passage at rest—our study is based on a distinct baseline population, namely patients undergoing acute reperfusion therapy. In this context, our findings extend the discussion on the value of TCD bubble testing as a screening tool by providing insights into its performance and relevance in a more severely affected stroke cohort.

Our findings demonstrate that the TCD-bubble test is a reliable screening tool for PFO detection, with a sensitivity of 97.1% and a negative predictive value of 97.4%, as confirmed by TEE. These values are consistent with previously published meta-analyses and prospective studies (12–16). The specificity of 71.2% and PPV of 69.4% in our cohort were slightly lower than in some earlier studies (13), likely reflecting TCD’s sensitivity for extracardiac/physiological shunts or pulmonary arteriovenous malformations (15). Given the minimal time difference between intracardiac and intrapulmonary shunts, as shown by Komar et al. (17), confirmatory TEE remains essential before therapeutic decisions such as PFO closure. In this context, TCD should be regarded as a highly sensitive screening modality rather than a stand-alone diagnostic tool (16).

Importantly, using ROC analysis, our findings indicate that a threshold of ≥3 bubbles provides a balanced diagnostic profile, with sensitivity of 91.4% and NPV of 93.9%. Applying this threshold reclassified 20% of TCD-positive cases (1–2 bubbles) as below the cut-off; however, confirmatory transesophageal echocardiography in this borderline subgroup still identified PFO in a subset of patients (16.7%), indicating that low-grade shunting does not reliably exclude PFO and should therefore not preclude further diagnostic evaluation in clinically appropriate cases. This quantitative approach complements prior predominantly qualitative strategies (12) and aligns with structured shunt grading concepts discussed by Palazzo et al. (16), including distinctions between binary International Consensus Criteria (classifying any detected microbubble as a positive RLS) and more granular systems such as the Spencer Logarithmic Scale. While our findings suggest that a ≥3-bubble threshold may improve specificity and help refine patient selection for further evaluation, this should be interpreted as supportive of individualized decision-making rather than a directive to omit invasive testing when <3 bubbles are detected. Given the sample size and internally derived ROC analysis, prospective validation in larger cohorts is required before firm clinical recommendations can be made.

Additionally, we analyzed the mode of shunt detection. Notably, in 66% of RLS-positive cases, microbubbles were detected at rest, suggesting a substantial proportion of large or persistent shunts. Although the prognostic relevance of resting versus provoked RLS in TCD remains uncertain, TEE-based studies indicate that PFOs visible at rest and those with high-risk anatomical features (e.g., marked septal mobility) are associated with increased stroke recurrence (18, 19). Consistent with this observation, our cohort showed a relatively high EVT rate of 39% among TCD bubble-tested patients. This may be attributable to the predominance of larger shunts, as reflected by the high proportion of bubble transit at rest, potentially facilitating the passage of larger emboli through the PFO and thereby contributing to large-vessel occlusion. This assumption is supported by anatomical considerations, as the mean diameter of a patent foramen ovale (~4.9 mm) exceeds that of major intracranial vessels such as the main trunk of the middle cerebral artery (~3 mm) and its cortical branches (~1 mm), allowing emboli of clinically relevant size to traverse the interatrial septum (20).

This study has several limitations. The retrospective single-center design and modest sample size limit generalizability. Many EVT-treated or intubated patients could not undergo TCD testing, leaving 83 patients for final diagnostic accuracy analyses. Not all TCD-tested patients received confirmatory TEE—particularly when TCD was negative or alternative etiologies were identified—introducing potential partial verification bias. In addition, TEE was not systematically blinded to TCD findings, creating a risk of diagnostic review bias. Sedation during TEE may impair Valsalva performance and lead to underdetection of PFO. Sedation-related limitations of TEE may be reduced through optimized minimal-sedation protocols (using topical pharyngeal anesthesia or minimal, short-acting sedation) and smaller probes, while a multimodal diagnostic strategy using non-sedated first-line imaging (e.g., transthoracic echocardiography, cardiac CT, or MRI) can reserve TEE for confirmatory assessment and help minimize cooperation-related false negatives.

Although TCD-bubble testing followed standardized protocols, dual-gate systems and automated bubble counting were not routinely available, so manual quantification may have introduced observer variability. Finally, inclusion was restricted to patients undergoing IVT and/or EVT, resulting in a cohort enriched for more severe strokes and potentially higher PFO prevalence, with more frequent competing etiologies such as atrial fibrillation or macroangiopathy. Thus, findings may not be generalizable to patients with TIA or minor stroke but primarily apply to AIS patients requiring reperfusion therapy. Prospective multicenter studies are warranted to validate quantitative thresholds and TCD-guided diagnostic strategies.

## Conclusion

In patients aged ≤65 years with AIS undergoing reperfusion therapy, TCD-bubble testing represents a highly sensitive and clinically valuable screening modality for PFO detection, and a quantitative threshold of ≥3 microbubbles may refine diagnostic stratification, although confirmatory TEE remains indispensable before therapeutic decision-making.

## Data Availability

The original contributions presented in the study are included in the article/Supplementary material, further inquiries can be directed to the corresponding author.

## Footnotes

1
https://www.ibm.com/products/spss
