# Supplementary material for: Bubble-test for detection of a patent foramen ovale in young to middle-aged ischemic stroke patients
Source: Front Neurol. 2026 May 11;17:1743561. doi: 10.3389/fneur.2026.1743561 (PMC13199003; doi:10.3389/fneur.2026.1743561)
Supplement: Supplementary file 1 [file Data_Sheet_1.pdf]

Supplementary Material

Manuscript: **Bubble-Test for detection of a patent foramen ovale in young ischemic stroke patients: Subanalysis of the STAMINA registry**

Content:

sFigure 1: RLS-Status depending on age

sFigure 2: Distribution of functional outcome at 90d comparing patients with detected RLS versus those without

sTable 1: Differences and Characteristics according to RLS status (RLS vs No RLS)

sTable 2: Comparison of AIS patients who received or did not receive TEE

sMethods: A: TCD-protocol

B: TEE-protocol

sFigure 1: RLS-Status depending on age

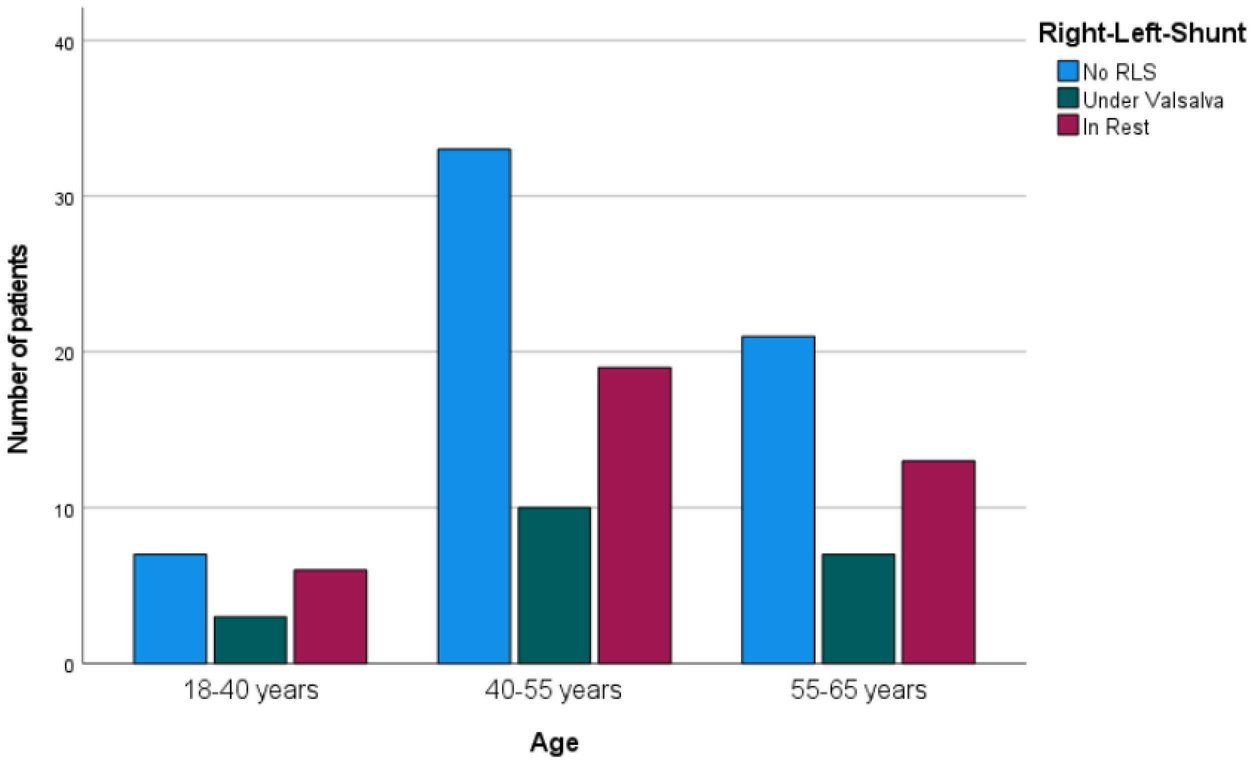

Age distribution of AIS-patients according to their RLS-status. The bars represent the absolute number of patients depending on their RLS-Status: color blue= no RLS, colour green = RLS detected under Valsalva maneuver, colour purple = RLS detected in rest; x-axis shows the age distribution

Abbreviations: RLS, Right-Left-Shunt

sFigure 2: Distribution of functional outcome at 90d comparing patients with detected RLS versus those without

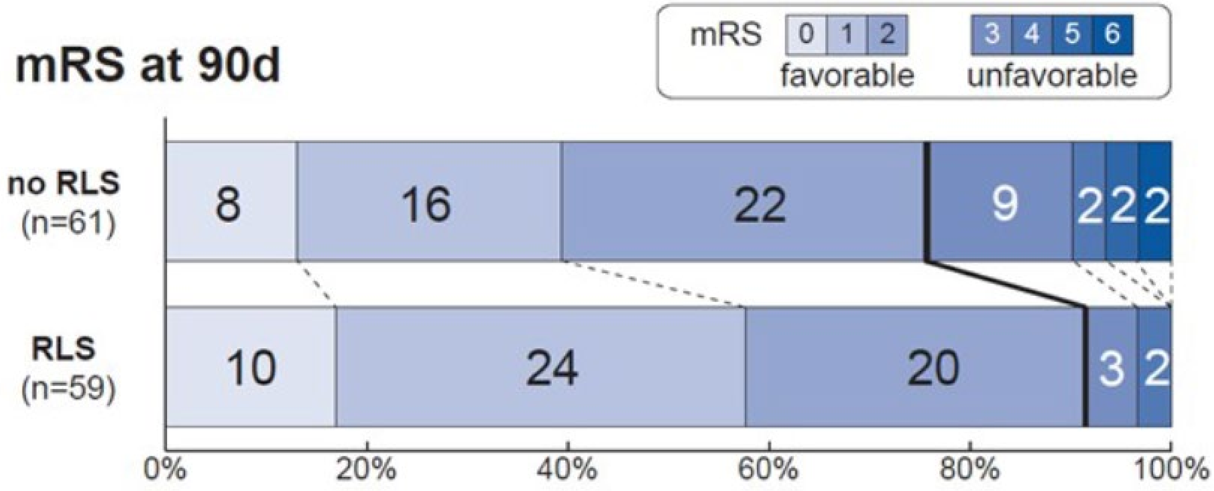

Distribution of mRS at 90 days comparing patients with RLS and without RLS. The bars represent the absolute number of patients and the scale indicates the percentage of patients. The bold line separates favorable (mRS, 0–2) and unfavorable outcome (mRS, 3–6).

Abbreviations: RLS, Right-Left-Shunt; mRS, modified Rankin Scale

sTable 1: Differences and Characteristics according to RLS status (RLS vs No RLS)

**AIS-patients ≤ 65a with Bubble Test (n=120)**	**RLS** (n=59)	**No RLS** (n=61)	**P Value**
Age, y; median (IQR)	53 (46-57)	52 (48-58)	0.71
Female sex; n (%)	17 (29%)	20 (33%)	0.64
Body weight; median (IQR)	81 (73-95)	80 (67-95)	0.46
**Prior medical history; n (%)**			
Premorbid mRS; median (IQR)	0 (0-0)	0 (0-0)	0.74
Alcohol abuse	6 (10%)	8 (13%)	0.58
Nicotine abuse	29 (49%)	29 (48%)	0.48
Hypertension	28 (47%)	38 (62%)	0.08
Diabetes mellitus Type II	8 (14%)	9 (15%)	0.82
Hypercholesterolemia	38 (64%)	31 (51%)	0.16
Coronary artery disease	5 (8%)	7 (11%)	0.56
Atrial fibrillation	1 (2%)	5 (8%)	0.22
Renal failure	1 (5%)	7 (11%)	0.20
**Stroke characteristics**			
NIHSS on admission; median (IQR)	5 (3-10)	6 (3-11)	0.45
GCS on admission: median (IQR)	15 (14-15)	15 (13-15)	0.21
TOAST classification; n (%)			0.001
- Microangiopathy<br>- Macroangiopathy<br>- Cardioembolic<br>- Unknown<br>- Other	3 (5%)<br>2 (3%)34<br>1 (2%)<br>36 (61%)<br>17 (30%)	6 (10%)<br>6 (10%)<br>7 (11%)<br>40 (66%)<br>2 (3%)	
Arteriosclerosis in Duplex Sonography; n (%)	33 (56%)	36 (60%)	0.007
TEE	45 (76%)	38 (62%)	0.19
PFO	34 (58%)	1 (1,6%)	0.001
**Outcome Scores**			
NIHSS at discharge; median (IQR)	1 (0-3)	2 (1-5)	0.011
mRS 90 days; median (IQR)	1 (1-2)	2 (1-3)	0.024

Abbreviations: RLS, Right-Left-Shunt; IQR, interquartile range; mRS, modified Rankin Scale; NIHSS, National Institutes of Health Stroke Scale; GCS, Glascow Coma Scale, TEE, transesophageal echocardiography; PFO, patent foramen ovale

sTable 2: Comparison of AIS patients who received or did not receive TEE

**AIS-patients ≤ 65a treated with IVT/EVT (n=383)**	**TCD-Bubble Test + TEE** (n=83)	**TCD without TEE** (n=37)	P **Value**
Age, y; median (IQR)	50 (44-57)	55 (52-61)	0.001
Female sex; n (%)	25 (30%)	12 (32%)	0.80
Bodyweight, kg, median (IQR)	80 (70-95)	80 (69-95)	0.98
**Prior medical history; n (%)**			
Premorbid mRS; median (IQR)	0 (0-0)	0 (0-0)	0.44
Alcohol abuse	8 (10%)	5 (14%)	0.24
Nicotine abuse	35 (42%)	16 (44%)	0.64
Hypertension	41 (49%)	25 (67%)	0.043
Diabetes mellitus Type II	10 (12%)	7 (19%)	0.29
Hypercholesterolemia	47 (57%)	22 (61%)	0.65
Coronary artery disease	7 (8%)	5 (14%)	0.36
Atrial fibrillation	4 (1%)	2 (6%)	0.81
Post-ischemia	8 (10%)	4 (11%)	0.80
**Stroke characteristics**			.
NIHSS on admission; median (IQR)	5 (3-11)	5 (3-8)	0.83
GCS on admission: median (IQR)	15 (13-15)	15 (13-15)	0.23
TOAST classification; n (%)			0.07
- Microangiopathy<br>- Macroangiopathy<br>- Cardioembolic<br>- Unknown<br>- Other	5 (8%)<br>3 (5%)<br>5 (8%)<br>32 (52%)<br>17 (27%)	4 (15%)<br>5 (19%)<br>3 (11%)<br>13 (48%)<br>2 (7%)	
Endovasular thrombectomy; n (%)	31 (38%)	16 (43%)	0.57
Intravenous thrombolysis; n (%)	73 (88%)	32 (87%)	0.82
PFO	31 (38%)	4 (11%)	0.06
**Outcome Scores**			
NIHSS at discharge; median (IQR)	1 (0-4)	4 (1-10)	0.29
mRS 90 days; median (IQR)	1 (1-2)	2 (1-2)	0.25

Abbreviations: AIS, acute ischemic stroke; IVT, intravenous thrombolysis; IQR, interquartile range; mRS, modified Rankin Scale; NIHSS, National Institutes of Health Stroke Scale; GCS, Glascow Coma Scale

sMethods

A) TCD-Bubble test protocol:

Examiner:
The TCD-Bubble test was performed by two certified neurologists (one with min. 3 months experience).

Device:
The middle cerebral artery was insonated using an ACUSON Sequoia Select (Siemens Healthineers).

Patients position:
The patient is in a supine position, the arm used for injection in the horizontal position with a 20-gauge in the right cubital vein

Preparation, choice and injection of the contrast agent:
A contrast suspension was prepared by using a three-way stopcock connected to a 10-ml syringe 1 with a mixture of 2 mL of the patient's venous blood,with 4 mL isotonic saline and to syringe 2 with 0.5 mL air and connected to a short tube leading from the antecubital vein. The blood/saline mixture is quickly exchanged with the air mixture for at least ten times. The patients blood stabilizes the microbubbles for 30-60s (Gentile et al.2014). After preparation the contrast agent is injected directly as a bolus into the vein. Injections were performed (1) at rest and (2) 4–8 seconds before a standardized Valsalva maneuver, with ≥1 minute between injections.

Valsalva maneuver:
Vaslsalva maneuver was explained and trained before performing a TCD-Bubble test. The instruction contained holding the breath and then pressing for at least 5 seconds (Widder et al. 2018).

Quantitative Assessment of RLS:

Microembolic signals ("bubble hits") were recorded continuously, and the number and temporaldistribution were documented. Right-left shunt was scored according to the following criteria:

– **"Clear evidence of RLS"** was defined as detection of bubbles at rest or >20 bubbles during the Valsalva maneuver.

– **"Minor evidence of RLS"** was defined as detection of ≤20 bubbles during the Valsalva maneuver only.

B) TEE protocol:

Transesophageal echocardiography was performed in sedation (with Midazolam and/or Propofol) after written informed consent. We used a GE 6Tc-RS probe and a vivid S70 ultrasound machine from GE HealthCare (https://www.gehealthcare.de/). Recorded loops were viewed and reanalyzed after the examination in the analyzing tool view point 6. View of left and right atrium was established by a midesophageal location in a range of 50 degrees (short axis) to 110 degrees (bicaval view). Blood attenuated saline was injected and Valsalva maneuver performed by an assistant by manually increasing abdominal pressure. PFO was defined as large if transfer from right to left atrium exceeded 25 bubbles within 3 heart cycles.

TEE was planned unless one or more of the following exclusion criteria were present: (1) clinical instability or medical contraindication to TEE, (2) patient refusal or lack of informed consent, (3) discharge or inter-hospital transfer before examination, (4) logistical or capacity-related constraints despite formal TEE request, (5) establishment of a definite alternative stroke etiology rendering further PFO diagnostics clinically unnecessary, or (6) previously known PFO.
